# Psychometric evaluation of the Chinese version of the Herth Hope Index (HHI) in Chinese children with cancer

**DOI:** 10.1038/s41598-023-33838-0

**Published:** 2023-04-26

**Authors:** Qi Liu, John Wai-Man Yuen, Ka-Yan Ho, Katherine-Ka-Wai Lam, Winsome Lam, Huilin Cheng, Dong Liang Yang, Shirley-Siu-Yin Ching, Frances-Kam-Yuet Wong

**Affiliations:** 1 School of Nursing, Hong Kong Polytechnic University, Hung Hom, Hong Kong SAR China; 2 Cangzhou Medical College, China, Cangzhou, China

**Keywords:** Patient education, Quality of life

## Abstract

Hope plays an extremely important role in protecting childhood cancer patients from psychological distress caused by cancer. The availability of a valid and reliable instrument that can accurately assess hope is crucial for the development of interventions to enhance hope among childhood cancer patients. This study aimed to examine the psychometric properties of the Chinese version of the Herth Hope Index (HHI). Chinese childhood cancer patients aged 8–17 years (n = 412) were invited to participate in this cross-sectional study. Participants completed the Chinese translated version of the HHI, the Center for Epidemiology Studies Depression Scale for Children and the Paediatric Quality of Life Inventory 3.0 Cancer Module. Exploratory factor analysis and confirmatory factor analysis were conducted to assess the structural validity of the HHI. Content validity, convergent validity, internal consistency, and test–retest reliability at 2 weeks were also examined. The content validity index for items ranged from 0.8 to 1.0, and that for the scale was 0.9, demonstrating appropriate content validity. There was a positive correlation between HHI and Center for Epidemiology Studies Depression Scale for Children scores and a negative correlation between HHI and Paediatric Quality of Life Inventory 3.0 Cancer Module scores. The results indicated that the Chinese version of the HHI showed reasonable convergent validity and discriminant validity. Exploratory factor analysis yielded a three-factor model, which could explain 82.74% of the total variance. The confirmatory factor analysis results showed that χ^2^/df was 2.20, comparative fit index was 0.98, goodness of fit index was 0.94, and root-mean-square error of approximation was 0.07. Cronbach’s alpha was 0.78, indicating good internal consistency. The findings of the study showed that the Chinese version of the HHI (11-item) is a reliable and valid instrument for assessing hope among Chinese childhood cancer patients. Evidence-based interventions can be provided to enhance hope in this population.

## Introduction

Cancer is a leading cause of death among children, and 400,000 new cases of childhood cancer are diagnosed worldwide each year^1^. In China, approximately 22,000 children are newly diagnosed with cancer every year^2^. According to Lu et al.^3^, leukaemia is the most common type of childhood cancer, followed by brain tumour and lymphoma. Breakthroughs in medical treatment have increased the chance of survival to more than 80% for most types of childhood cancer^4,5^. Despite these improved survival outcomes, medical treatment remains a highly stressful event for children with cancer, which significantly affects their psychological well-being^4^. High levels of depression, anxiety and fear are the most common problems reported by children during cancer treatment^6^. In a cross-sectional study of 98 hospitalised Chinese children with cancer, approximately 63% were at risk of depression^7^. Additionally, short interviews with 89 Chinese children revealed that different degrees of sadness and worry were attributable to unclear prognoses, invasive medical procedures, and long-term hospitalisation^6^. The psychological impact of cancer on a child should never be overlooked or underestimated. Numerous studies have shown that these psychological symptoms can activate somatic pathways and thus aggravate common physical symptoms induced by cancer, such as pain, nausea and fatigue^8^. Additionally, some psychological symptoms, particularly anxiety and fear, may lead to poor compliance with medical treatment and impede recovery^9,10^.

Increasing evidence indicates that hope plays an important protective role against psychological distress caused by life-threatening conditions such as childhood cancer^11^. In adults, hope is defined as a confident but uncertain expectation of a future good that appears realistically possible and is personally significant to the individual^12^. This concept is also relevant for children. Previous research indicates that children begin to develop the concept of hope from 7 years of age^13^. In 2000, Snyder defined hope in children as a cognitive set involving a belief in one’s ability to produce workable routes to goals, as well as self-related beliefs about initiating and sustaining movement along these routes towards the desired goals^13^. Previous studies suggested that hope can transform the life of a child and their family members during their most difficult challenges^13^. A sense of hope can reduce despair in the face of life-threatening medical conditions, enrich the human experience with strength and joy, and cultivate a child’s resources to cope with adversity^13^. Given these benefits, the assessment of hope in children with cancer is crucial for gaining a thorough understanding of their responses to stress and adversity, which in turn, facilitates the development of appropriate interventions that enhance children’s ability to cope with the psychological distress resulting from diagnosis and medical treatment throughout their cancer journey. The protective role of hope in psychological distress has been confirmed in multiple studies of adult cancer patients, with higher levels of hope being associated with better quality of life (QoL)^14^. However, there is a paucity of similar evidence from studies of children with cancer. Most existing literature has explored the concept of hope among parents, siblings, and caregivers of children with cancer^15^.

To date, no study has measured hope in Chinese children with cancer or examined how this factor affects their psychological well-being. Some evidence suggests that culture has an important effect on the perception of hope. For example, hope is considered to be a theological virtue in Western cultures^16^. In contrast, many Chinese people are influenced by Confucianism, which emphasises fatalism and often regards hope as fighting against challenges that are designated as fate^17^. Given this difference, a reliable and valid instrument for accurately measuring hope in children with cancer, particularly those in the Chinese context, is necessary and important.

A comprehensive literature review identified four instruments that can be used to measure the level of hope in children. The most promising of these measures is the Herth Hope Index (HHI). This instrument was initially developed for adults with acute, chronic or terminal conditions, and is based on a multi-dimensional concept of hope developed from philosophical, theological, sociological, psychological, and nursing perspectives^18^. The HHI contains three subscales, each corresponding to one of the three domains of hope: (1) inner sense of temporality and future, defined as the perception of a desired outcome that can be realistically achieved in the near or distant future; (2) inner positive readiness and expectancy, defined as confidence in one’s ability to effect a plan and achieve the desired outcome; and (3) inter-connectedness with the self and others, defined as the recognition of interdependence and interconnectedness between the self and others, as well as between the self and the spirit^18^. Herth examined the psychometrics of the HHI in a convenience sample of 192 adults^18^. The HHI was found to have excellent internal consistency and test–retest reliability and appropriate concurrent criterion-related, divergent, and construct validity^18^.

The HHI was previously used to measure hope in children with different chronic medical conditions, including asthma and cystic fibrosis^11^. The applicability of this instrument to paediatric patients is supported by a previous study, which indicated that most items of the HHI reflected the phrasing used by children to describe the concept of hope^11^. The psychometric appropriateness of the HHI as a measure of hope in children with cancer was demonstrated in a Western study of 201 paediatric oncology patients^11^. The results of that study generally indicated the reliability and validity of the HHI and indicated good internal consistency and appropriate discriminant and convergent validity. Although the HHI is available for paediatric use in Western countries^11^, it has never been translated into Chinese, and its psychometric properties have never been tested in a Chinese context. Children living in this context differ considerably from Western children in terms of culture, particularly in terms of their perceptions of the nature and meaning of their illness, as well as their hopes regarding diagnosis and treatment^16,17^. Therefore, items that are appropriate for Western children may not be appropriate for Chinese children^19^. Accordingly, the relevancy and psychometric properties of the HHI should be evaluated before applying this instrument to Chinese children with cancer. This study aimed to translate and validate an instrument for the accurate assessment of hope in Chinese children with cancer and thus bridge the gap in the existing literature. The study objective was to examine the psychometric properties of the Chinese version of the HHI. In addition, we examined the factorial structure of the HHI using exploratory factor analysis (EFA) and confirmatory factor analysis (CFA).

## Methodology

### Study design

We used a cross-sectional study to translate and validate the Chinese version of the HHI at Hunan Children Hospital, South-central China. This hospital was chosen because it is a key hospital in the province that provides treatment for paediatric oncology patients.

### Subjects

Children who met the following inclusion criteria were invited to participate in this study: (1) aged 8–17 years, (2) able to speak either Cantonese and/or Mandarin and read Chinese, and (3) having a confirmed diagnosis of cancer and currently receiving active treatment. Children younger than 8 years old were not invited to participate because they might not be able to understand the concept of hope^13^. Children with evidence of a second malignancy or recurrence and those whose medical records indicated cognitive and behavioural problems were excluded.

There are no clear rules for calculating sample sizes for factor analyses. However, larger samples are recommended if the data are not normally distributed^20^. Dixon suggested that at least 10 subjects per item should be included when performing EFA^21^. Because the HHI contains 12 items, at least 120 subjects is necessary for conducting EFA^21^. Concerning CFA, a sample of around 200 subjects is required^22^. Hence, at least 320 subjects therefore were required in this study. In addition, we also calculated the sample size using the correlations between HHI and other instruments. We calculated the sample size using G*Power 3.1. According to Cohen^23^, f^2^ = 0.02 is considered a “small” effect size, 0.15 represents a “medium” effect size and 0.35 a “large” effect size. According to previous studies^24,25^, the effect sizes of our proposed correlations between HHI and other instruments ranged from medium to large. We therefore proposed a medium -to- large size (f^2^ = 0.25) for the correlations. To achieve power of 0.95 and α of 0.05, a minimum sample size of 195 was required. After a comparison of sample calculations using both methods, it was decided to use the larger sample size of 320.

### Translation of the HHI

The process of translation was based on the guidelines proposed by the World Health Organization (http://www.who.int/substanceabuse/researchtools/translation/en/). The whole process aimed to achieve cross-cultural and conceptual equivalence, rather than linguistic and literal equivalence. The process included six steps: (1) forward translation, (2) expert panel, (3) back translation, (4) pre-testing and cognitive interviewing, (5) final version, and (6) documentation.

Initially, a paediatric nurse specialist familiar with the concept of hope translated the HHI from English to Chinese. An expert panel then identified and resolved any inadequate expressions in the tentative translated version. The panel included one professor, two assistant professors, one research assistant professor, one paediatric oncologist, and one paediatric oncology ward manager. All panel members have extensive knowledge about conducting studies with paediatric oncology patients and testing the psychometric properties of translated instruments. Next, another independent bilingual translator who was blinded to the original items was asked to translate the tentative Chinese version back into English. The retranslated English version was then compared with the original English version to ensure that the original meaning of each item has been maintained. The panel then discussed any discrepancy until a satisfactory version was reached. In a pilot study, the drafted Chinese version was applied to 10 Chinese children to ensure its comprehensibility. The finalised Chinese version of the HHI was the product of the above-described iterations. The whole process was documented to ensure that all procedures were traceable.

### Measures

#### The Chinese version of the Herth Hope Index (HHI)

This instrument was used to measure the levels of hope exhibited by our subjects. The instrument comprises 12 items categorised into three subscales: (1) temporality and future, (2) positive readiness and expectancy, and (3) interconnectedness. Subjects were asked to rate each item on a four-point Likert scale. Possible scores ranged from 12 to 48, with higher scores representing higher levels of hope^18^. Previous psychometric testing of the English version of the HHI have verified its reliability and validity for measuring hope among paediatric population, with the Cronbach’s α = 0.88^26^.

#### The Chinese version of the Centre for Epidemiological Studies Depression Scale for Children (CES-DC)

This scale was used to assess the number of depressive symptoms exhibited by each subject. Subjects were asked to rate each of the 20 items using a four-point Likert scale (0 = not at all; 1 = a little; 2 = some; 3 = a lot). Possible scores ranged from 0 to 60, with higher scores representing more depressive symptoms. The psychometric properties of this scale have been reported to have good internal consistency, with Cronbach’s α = 0.82, which supported the validity and reliability of this instrument for detecting depressive symptoms in Chinese children with cancer^6^.

#### The Chinese version of the Paediatric Quality of Life Inventory 3.0 Cancer Module (PedsQL 3.0)

The PedsQL was used to assess subjects’ QoL. This scale comprises 27 items categorised into eight domains: pain and hurt (two items), nausea (five items), procedural anxiety (three items), treatment anxiety (three items), worry (three items), cognitive problems (five items), perceived physical appearance (three items), and communication (three items). Subjects were asked to rate how often they have experienced each problem during the past 1 month, using a 5-point Likert scale (0 = never a problem; 1 = almost never a problem; 2 = sometimes a problem; 3 = often a problem; 4 = almost always a problem). Each item was transformed linearly to a scale of 0–100 (0 = 100; 1 = 75; 2 = 50; 3 = 25; 4 = 0). The overall scale score was then calculated as an average of the total item scores, with higher overall scores indicating better QoL^5^. This scale has been widely used in previous studies of Chinese children with cancer, and has adequate construct validity and good internal consistency (Cronbach’s α = 0.87) and test–retest reliability (0.79)^5^.

### Data collection

Prior to study commencement, ethical approval (HSEARS20220127001) was sought from the relevant Institutional Review Board at Hong Kong Polytechnic University. A poster containing the study’s details was posted on a noticeboard inside the paediatric oncology ward. Children who were interested in participating were able to contact the nurse-in-charge. Additionally, a research assistant approached children in the ward and ascertained their interest and willingness to participate. Written consent was sought from children’s parents after they received an explanation of the study’s details. Children were invited to sign their names on a child assent form. Children and parents received assurance that all provided information was kept strictly confidential, and a guarantee that refusal to participate would have no effect on the care received. The methods were carried out in compliance with the approved guidelines. All procedures performed in the studies were in accordance with the Helsinki declaration of 1964.

### Data analysis

#### Semantic and content equivalence

The expert panel assessed the semantic and content equivalence of the Chinese version of the HHI. For semantic equivalence, the panel members were asked to rate each translated item on a four-point Likert scale (from 1 = not equivalent to 4 = most equivalent). Any item that received a rating of either 1 or 2 from more than 20% of the panel members was amended. Regarding content equivalence, the panel members rated each item on a four-point Likert scale (from 1 = not relevant to 4 = most relevant). The content validity index (CVI) was calculated as the proportion of items that achieved a relevance rating of either 3 or 4 by all panel members. A CVI score of ≥ 0.9 indicates good content validity^27^.

#### Reliability testing

The internal consistency of the Chinese version of the HHI was assessed by calculating Cronbach’s alpha using the sample of 412. A value of ≥ 0.7 indicates acceptable reliability^28^. To evaluate test–retest reliability, 50 subjects were invited to respond to the Chinese version of the HHI 2 weeks later during follow-up telephone calls. The intra-class correlation coefficient (ICC) was calculated with the whole data set, and a value of ≥ 0.75 indicates an appropriate instrument for use in research^29^.

#### Factor structure

The total data (n = 412) were randomly split into data set A (n = 206) and data set B (n = 206). To examine the underlying factor structure of the Chinese version of the HHI, EFA was first performed with the data set A using the SPSS 26.0 for Windows (SPSS Inc., Chicago, IL, United States). Prior to EFA, we performed the Bartlett’s Test of Sphericity and the Kaiser–Meyer–Olkin Test to confirm that the data were adequate for EFA. The EFA was then conducted to extract the factors by using the Varimax orthogonal rotation method. The criterion for appropriate factor extraction was factor loading of 0.40 or higher^30^. In addition, there should be no cross-loading of items (i.e., loadings of 0.32 or higher on more than one factor) and no factors with fewer than three items^30^.

CFA was conducted with the data set B using LISREL version 8.8 for Windows (Scientific Software International Inc, Lincolnwood, IL, USA). Initially, we performed Bartlett’s Test of Sphericity and the Kaiser–Meyer–Olkin Test for Sampling Adequacy to confirm that the data were suitable for CFA. The overall fit between the data and the proposed factor structure was then assessed using different goodness of fit indices, including the chi-square to degrees of freedom (χ^2^/d.f.) ratio, root mean square error of approximation (RMSEA), comparative fit index (CFI), and goodness of fit index (GFI). The χ^2^/d.f. ratio assesses the global fit, with a value of < 3 indicating a good fit^31^. The RMSEA indicates the model fit according to the population discrepancy function, a standardised measure of approximation error. A value of < 0.05 indicates a good fit^31^. The GFI assesses the global fit between a theoretical model and the data. A value of ≥ 0.95 indicates a good model-data fit^31^. The CFI indicates the superior fit of the proposed model in comparison to an independence model. A value of ≥ 0.95 indicates a good fit^31^.

#### Convergent validity

Depression and QoL were set as comparative constructs. Pearson’s product-moment correlation coefficients between HHI scores and CES-DC (depression) scores, as well as between HHI scores and PedsQL (QoL) scores were calculated with a sample of 412. Previous literature indicated a negative correlation between hope and depression^24^ and a positive correlation between hope and QoL^25^. In addition, the average variance extracted estimate (AVE) value, which is the sum of squared loadings divided by the number of items^32^, was used to prove that each factor was consistently and accurately measured (n = 206). AVE more than or equal to 0.5 confirms the convergent validity^32^.

The AVE was analysed using the R programming language's semTools packages^33^.

#### Discriminant validity

The discriminant validity of the factors was examined using the Fronell-Larcker criterion^32^. According to this criterion, the square root of the AVE by a factor must be greater than the correlation between the factor and any other factor (n = 206).

### Ethical approval

This study has been approved by ethical approval Institutional Review Board at Hong Kong Polytechnic University (HSEARS20220127001).

## Results

### Demographic and clinical characteristics

We successfully recruited a total of 412 subjects between Jan 2022 and May 2022. Table 1 shows their demographic and clinical characteristics. The mean age of our subjects was 12.3 years (standard deviation [SD] = 2.9) and their mean household size was 4.14 (SD = 1.1). Of the recruited subjects, 56.3% (n = 232) were girls, 86.8% (n = 357) were non-religious, 52.2% (n = 215) of the children’s parents attained upper secondary school education, 66.8% (n = 275) were diagnosed with non-solid tumour, 52.9% (n = 232) were diagnosed within 6 months, and 20.8% (n = 86) had received multiple treatments for cancer.

**Table 1:** Sociodemographic and clinical characteristics of the participants (N = 412).

	Number (%)
Age range	
8–12 years	210 (51.0)
13–18 years	202 (49.0)
Gender	
Male	180 (43.7)
Female	232 (56.3)
Parents’ educational attainment	
Lower secondary school or below	197 (47.8)
Upper secondary school or above	215 (52.2)
Household Size	
1–3	149 (36.2)
4–5	226 (54.9)
> 5	37 (9.0)
Diagnosis	
Non-solid tumor	275 (66.8)
Solid tumor	137 (33.2)
Time since diagnosis	
< 6 months	218 (52.9)
6–12 months	102 (24.8)
> 1 year	92 (22.3)
Treatment received	
Surgery	41 (10)
Chemotherapy	259 (62.9)
Bone marrow transplant	26 (6.3)
Mixed method	86 (20.8)
Home religious’ affiliation	
With religion	55 (13.2)
No religion	357 (86.8)

### Validity

#### Semantic equivalence

The sematic equivalence for the translated scale was 0.916, with that for the items ranging from 0.8 to 1.0. These results suggested that the translated version was equivalent to the English version conceptually and equivalently.

#### Content validity

The I-CVIs values ranged from 0.8 to 1.0 and S-CVI was 0.9. These results indicated that the Chinese version of the HHI had satisfactory content validity.

#### Exploratory factor analysis

The Kaiser–Meyer–Olkin value was 0.87, and the Bartlett’s test of sphericity results were statistically significant (*p* < 0.001) revealing that the data collected by the translated HHI met the criteria for factor analysis. Table 2 shows the results of the EFA (n = 206). The three factors with an eigenvalue higher than 1 were identified. Because one item (“I feel scared about my future”) was found to be cross-loaded in different factors, it was removed from the scale. After removing this item, the EFA results of the 11-item HHI with a three-factor structure suggested that all of the factor loadings in each factor ranged from 0.677 to 0.879 and that the structure supported scale construction. Factor 1 (“inner sense of temporality and future”), explained 24.77% of the variance; factor 2 (“inner positive readiness and expectancy”) explained 27.30% of the variance; and factor 3 (“inter-connectedness with self and others”) explained 30.68% of the variance. The interpretation of the aforementioned three components was consistent with the proposed factor structures of the original English version of the HHI.

**Table 2:** Exploratory Factor Analysis of the Chinese version of HHI Scale (n = 206).

Items	12-item 3-factor model			11-item 3-factor model		
	Component 1	Component 2	Component 3	Component 1	Component 2	Component 3
I have a positive outlook toward life	0.850			0.861		
I have short, intermediate and/or long-range goal	0.876			0.879		
**I feel scared about my future**	0.515	0.538		–	–	–
I believe that each day has potential	0.809			0.811		
I feel all alone		0.670			0.677	
I have a faith that gives me comfort		0.862			0.865	
I have deep inner strength		0.809			0.813	
I am able to give and receive caring/love		0.879			0.883	
I can see a light at the end of the tunnel			0.814			0.808
I can recall happy/joyful times			0.858			0.854
I have a sense of direction			0.771			0.785
I feel my life has value and worth			0.775			0.788
Variance explained (%)	24.72	27.32	28.23	24.77	27.30	30.68
Total variance explained (%)			80.27			82.74

Component 1, Inner sense of temporality and future; Component 2, Interconnectedness with self and others; Component 3, Inner positive readiness and expectancy.

Cross-loaded item is in bold.

#### Confirmatory factor analysis

Table 3 shows the results of the CFA of the Chinese version of the HHI (n = 206). Various fit indices were used to evaluate the overall fits of the 12- and 11-item models of the Chinese version of the HHI on the basis of three factors. The results suggested that the 11-item model had a better fit index than the 12-item model.

**Table 3:** Fit statistics for the Chinese version of the HHI (n = 206).

Factor model	χ^2^/df	CFI	GFI	RMSEA
12-item 3-factor model	3.38	0.95	0.89	0.11
11-item 3-factor model	2.20	0.98	0.94	0.07

χ^2^/df, Chi-Square Mean/Degree of Freedom; CFI, Comparative Fit Index; GFI, Goodness of Fit Index; RMSEA, Root Mean Square Error of Approximation. Acceptable overall fit of each model was evaluated using the following indices: χ^2^/df: 3.00 or lower, CFI: 0.9 or higher, GFI: 0.9 or higher, RMSEA: 0.08 or less.

**Figure 1:**
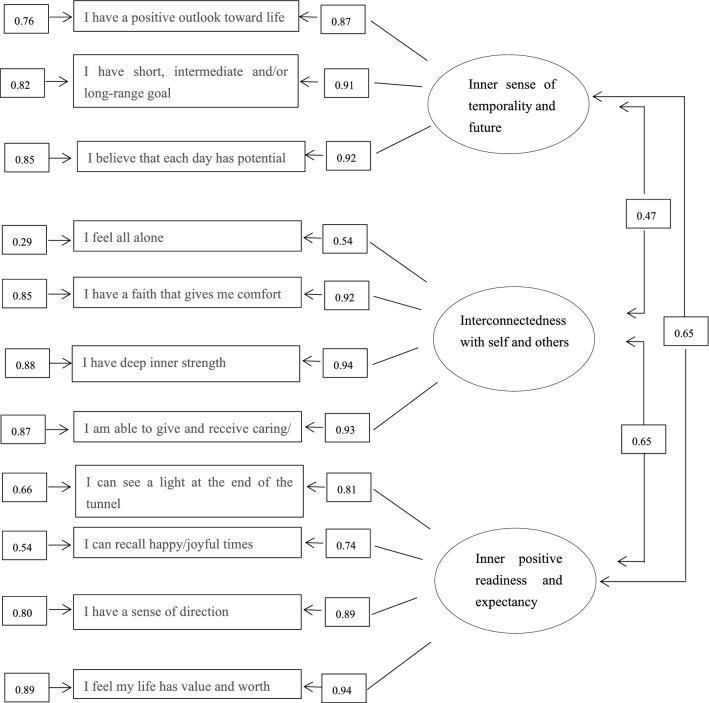
Confirmatory factor analysis for the Chinese version of 11-item 3-factor HHI (n = 206). [Three factors: Inner sense of temporality and future, Interconnectedness with self and others, and Inner positive readiness and expectancy. All the paths were significant in this model (*P* < 0.05).]

The parameter estimates of the 11-item three-factor model are shown in Fig. 1. All correlation matrices were less than 1 and were positively definite, indicating that the parameter estimates were reasonable. The factor loadings for each observed variable were high, ranging from 0.54 to 0.94. The t-values of all variables were greater than 1.96, suggesting statistically significant loadings.

#### Convergent validity

Spearman’s correlation coefficients of 0.60 and 0.33 were found between HHI and CES-DC scores (n = 412), and between HHI and PedsQL scores (n = 412), respectively. The results indicated that the translated version of the HHI had appropriate construct validity. The AVE values for the three factors (“inner sense of temporality and future”, “inner positive readiness and expectancy” and “inter-connectedness with self and others”) were 0.804, 0.726, 0.719, respectively, showing good convergent validity (n = 206).

#### Discriminant validity

For discriminant validity (n = 206), the square root of the AVE by all factors (“inner sense of temporality and future”, “inner positive readiness and expectancy” and “inter-connectedness with self and others”) were greater than the correlation between the factor and any other factor, suggesting adequate evidence for this validity (see Table 4).

**Table 4:** Factors discriminability (n = 206).

Factors	AVE	Factor 1	Factor 2	Factor 3
Factor 1	0.804	**0.897**		
Factor 2	0.726	0.495*	**0.852**	
Factor 3	0.719	0.656*	0.655*	**0.848**

Factor 1, Inner sense of temporality and future; Factor 2, Interconnectedness with self and others; Factor 3, Inner positive readiness and expectancy; AVE, Average variance extracted; *Correlation is significant at the 0.01 level (2-tailed); The bold is the square root of the AVE.

### Reliability

For the whole sample of 412, the Cronbach’s alpha coefficient for the internal consistency of the adapted scale was 0.78. The corrected item–total correlations ranged from 0.34 to 0.82, indicating acceptable internal consistency. The test–retest reliability coefficient (n = 50) at the 2-week interval was 0.82.

## Discussion

Hope is important for childhood cancer patients to cope with extreme distress resulting from cancer and its treatment^34^. A valid and reliable instrument for accurately assessing hope among childhood cancer patients is a prerequisite for the development of hope-based therapy. Previous studies have shown that the HHI is an appropriate instrument to assess hope in childhood cancer patients in Western countries. To bridge the gap in existing literature, this study translated and examined the psychometric properties of the Chinese version of the HHI in Chinese childhood cancer patients.

The Chinese version of the HHI showed high reliability, with an overall Cronbach’s alpha coefficient and ICC of 0.78 and 0.82, respectively. Hence, this instrument appears to be suitable for clinical and research applications in Chinese children with cancer.

Regarding convergent validity, we observed a negative correlation between CES-DC and HHI scores, and a positive correlation between PedsQL and HHI scores. The results are consistent with previous reports that childhood cancer patients who reported higher levels of hope had fewer depressive symptoms and better QoL^24,25^. Current results supported the satisfactory convergent validity of the Chinese version of the HHI.

In the current study, EFA yielded a three-factor structure with 12 items. This factor structure is consistent with that found by Herth in the original version of the HHI^18^. However, previous studies have reported varying factor structures comprising different items from the HHI. Benzein and Berg et al.^35^ suggested a two-factor structure with 12 items. Similarly, Wahl et al.^36^ identified a two-factor structure comprising 10 items. Phillips et al.^11^ proposed a one-factor structure with seven items. One possible reason for these varying factor structures in the HHI is that hope, as a measurable concept, appears be influenced by various factors, particularly the methodological approaches adopted by the studies as well as sample-dependent characteristics^37^.

Although our results supported a three-factor structure in the EFA, item 6 “I feel scared about my future” was cross-loaded on different factors and had a low factor loading relative to the other items. There are several potential explanations for this result. First, hope and fear are not mutually exclusive. A person can still be hopeful towards a situation in which they feel scared^11^. Second, this item used reverse scoring. Previous studies reported that reverse-scored items do not fit normal human logic, and people often make mistakes if they do not pay sufficient attention when responding^38–40^. Third, unlike children with cancer in the West, Chinese children are deeply influenced by Confucianism which emphasises fatalistic beliefs^41^. Hence, Chinese children with cancer generally believe that nothing can be done to change their situation, and tend to adopt emotion-focused strategies, particularly avoidance, to deny any negative thoughts in relation to cancer and imagine a positive future^41^. This is supported by the finding that 52.4% and 25.7% of respondents in our study answered “strongly disagree” and “disagree” regarding item 6, respectively. In addition, previous literature indicated that cross-loaded items will affect the discriminant validity of scales if these items are retained^42^. Given that item 6 was cross-loaded on different factors, as well as the low factor loading and irrelevance of this item in the Chinese cultural context, it was removed from the Chinese version of the HHI. After removing this item, the EFA showed higher total variance explained for the 11-item version than for the original 12-item version (82.74% vs. 80.27%, respectively). Additionally, our CFA results revealed that the 11-item structure achieved a better fit than the original 12-factor structure, providing further support for removing item 6.

CFA was performed on another half of the sample to test the validity of the structure obtained after EFA. According to the goodness-of-fit indices obtained in the current study, the three-factor structure with 11 items was confirmed, and hence we concluded that the factor structure underlying the HHI was appropriate for Chinese childhood cancer patients.

Although the three-factor structure was found to be appropriate for the translated HHI, the CFA results indicated that the factor loadings for item 2 (“I have short, intermediate and/or long-range goals”) and item 11 (“I believe that each day has potential”) on the “temporality and future” domain were higher than those obtained in a previous validation study of HHI among adolescent cancer patients in the United States (item 2: 0.91 vs 0.45; item 11: 0.92 vs 0.45)^11^. Higher factor loadings suggest that future expectations substantially contribute to the construction of hope for Chinese childhood cancer patients, unlike children in the West who mostly rely on religious belief and practices to obtain hope. This finding is also consistent with our previous qualitative study indicating that short-term aspirations (e.g., improvement in clinical status) and long-term aspirations (e.g., going back to school and resuming their normal life) were a major source of hope for Chinese childhood cancer patients, which further motivated them to continue cancer treatment and fight the disease^43^. The phenomenon described above could potentially be explained by the fact that most Chinese people are not religious^43^. Carson et al.^44^ defined two types of hope, the first is eternal hope that is anchored to a belief in God, and the second is existential hope that focuses on the future orientation of the individual. Because most Chinese people do not have religious beliefs^45^, existential hope is more common in China, and people rely more strongly on imagining their future to construct hope.

### Implications for future practice

A growing body of evidence indicates that hope can mitigate negative and/or promote positive psychological outcomes among children with cancer, affecting their QoL^24,25,46^. Given the important role of hope in psychological health, the validated HHI can be used as a tool for routinely measuring hope in Chinese childhood cancer patients, enabling early interventions to be provided. Hope-based therapy is commonly used for adult cancer patients^47^, but is less common among Chinese childhood cancer patients because of a lack of instruments for assessing their hope. The validated HHI can be used to guide the development of hope-based therapy in this population, particularly the evaluation of intervention effectiveness.

### Limitations

This study involved several limitations that should be considered. First, the sample only contained patients from one clinical setting, and the use of convenience sampling might limit the generalisability of the results. Additionally, most participants were diagnosed with cancer within 1 year, and those who were terminally ill were not recruited for this study because of ethical considerations. Further research should build on this work, examining the stability of the factor structure underlying HHI across a more diverse population.

## Conclusion

The current study demonstrated that the Chinese version of the HHI is a reliable and valid instrument for measuring hope in Chinese childhood cancer patients. The EFA and CFA results confirmed that the factor structure of the Chinese version was congruent with the proposed three-factor model of the original version. This instrument can be applied in future hope-related studies in Chinese childhood cancer patients, and may be useful for informing the development of evidence-based programmes to enhance hope in this population.

## Data Availability

The data will be available from the corresponding author upon reasonable request.

## Abbreviations

HHI: Herth Hope Index
QoL: Quality of Life
EFA: Exploratory Factor Analysis
CFA: Confirmatory Factor Analysis
CES-DC: Centre for Epidemiological Studies Depression Scale for Children
PedsQL 3.0: Paediatric Quality of Life Inventory 3.0 Cancer Module
CVI: Content Validity Index
ICC: Intra-class Correlation Coefficient
χ^2^/df: Chi-square to Degrees of Freedom
RMSEA: Root Mean Square Error of Approximation
CFI: Comparative Fit Index
GFI: Goodness of Fit Index

## References

[1] World Health Organization (2021). CureAll Framework: WHO Global Initiative for Childhood Cancer: Increasing Access Advancing Quality Saving Lives.

[2] Li Z (2021). Pediatric cancer surveillance in China: A hospital-based introduction. Pediatr. Investig..

[3] Li L, Huang C, Huang H (2015). Childhood cancer: An emerging public health issue in China. Ann. Transl. Med..

[4] Li HCW, Chung OKJ, Chiu SY (2010). The impact of cancer on children's physical, emotional, and psychosocial well-being. Cancer Nurs..

[5] Li HCW (2013). Confirmatory factor analysis of the Chinese version of the Pediatric Quality-of-Life Inventory cancer module. Cancer Nurs..

[6] William Li HC, Chung OKJ, Ho KY (2010). Center for epidemiologic studies depression scale for children: Psychometric testing of the Chinese version. J. Adv. Nurs..

[7] Li HC, Chung OK, Chiu SY (2010). The impact of cancer on children's physical, emotional, and psychosocial well-being. Cancer Nurs..

[8] Rodgers CC, Hooke MC, Hockenberry MJ (2013). Symptom clusters in children. Curr. Opin. Support. Palliat. Care.

[9] Pao M, Bosk A (2011). Anxiety in medically ill children/adolescents. Depress. Anxiety.

[10] Griffiths M, Schweitzer R, Yates P (2011). Childhood experiences of cancer: An interpretative phenomenological analysis approach. J. Pediatr. Oncol. Nurs..

[11] Phillips-Salimi CR, Haase JE, Kintner EK, Monahan PO, Azzouz F (2007). Psychometric properties of the Herth Hope Index in adolescents and young adults with cancer. J. Nurs. Meas..

[12] Moseley J (1985). Symposium on compassionate care and the dying experience. Alterations in comfort. Nurs. Clin. North Am..

[13] Snyder CR (2000). Handbook of Hope: Theory, Measures, and Applications.

[14] Mahendran R (2016). Biopsychosocial correlates of hope in Asian patients with cancer: A systematic review. BMJ Open.

[15] Bally JM (2014). Keeping hope possible: A grounded theory study of the hope experience of parental caregivers who have children in treatment for cancer. Cancer Nurs..

[16] Peterson, C. & Park, N. Classifying and Measuring Strengths of character. In *Oxford Handbook of Positive Psychology*, Vol. ii, 25–33 (2009).

[17] Wong MYF, Chan SWC (2006). The qualitative experience of Chinese parents with children diagnosed of cancer. J. Clin. Nurs..

[18] Herth K (1992). Abbreviated instrument to measure hope: Development and psychometric evaluation. J. Adv. Nurs..

[19] Ferraz M (1997). Cross cultural adaptation of questionnaires: What is it and when should it be performed?. J. Rheumatol..

[20] Hu L-T, Bentler PM, Kano Y (1992). Can test statistics in covariance structure analysis be trusted?. Psychol. Bull..

[21] Dixon J (2005). Exploratory factor analysis. Stat. Methods Health Care Res..

[22] Kline RB, Little TD (2016). Principles and practice of structural equation modeling. Methodology in the Social Sciences.

[23] Cohen J (2013). Statistical Power Analysis for the Behavioral Sciences.

[24] Germann JN, Leonard D, Heath CL, Stewart SM, Leavey PJ (2018). Hope as a predictor of anxiety and depressive symptoms following pediatric cancer diagnosis. J. Pediatr. Psychol..

[25] Rosenberg AR (2018). Hope, distress, and later quality of life among adolescent and young adults with cancer. J. Psychosoc. Oncol..

[26] Canada AL, Murphy PE, Fitchett G, Peterman AH, Schover LR (2008). A 3-factor model for the FACIT-Sp. Psychooncology.

[27] Lenz ER (2010). Measurement in Nursing and Health Research.

[28] Nunnally, J. C. An overview of psychological measurement. *Clin. Diagn. Mental Disord.* 97–146 (1978).

[29] Portney LG, Watkins MP (2009). Foundations of Clinical Research: Applications to Practice.

[30] Costello AB, Osborne J (2005). Best practices in exploratory factor analysis: Four recommendations for getting the most from your analysis. Pract. Assess. Res. Eval..

[31] Munro BH (2005). Statistical Methods for Health Care Research.

[32] Fornell, C. & Larcker, D. F. *Evaluating structural equation models with unobservable variables and measurement error* 18 thesis, American Marketing Association, (1981).

[33] Jorgensen, T. D., Pornprasertmanit, S., Schoemann, A. M., & Rosseel, Y. 2022. semTools: Useful tools for structural equation modeling. R package version 0.5–6 (accessed 3 March 2023); https://CRAN.R-project.org/package=semTools.

[34] Proserpio T (2020). Spirituality and sustaining hope in adolescents with cancer: The patients' view. J. Adolesc. Young Adult. Oncol..

[35] Benzein E, Berg A (2003). The Swedish version of Herth Hope Index–an instrument for palliative care. Scand. J. Caring Sci..

[36] Wahl AK (2004). The Norwegian version of the Herth Hope Index (HHI-N): A psychometric study. Palliat. Support. Care.

[37] Rustøen T, Lerdal A, Gay C, Kottorp A (2018). Rasch analysis of the Herth Hope Index in cancer patients. Health Qual. Life Outcomes.

[38] Salazar MS (2015). The dilemma of combining positive and negative items in scales. Psicothema.

[39] Sauro, J. & Lewis, J. R. in *Proceedings of the SIGCHI conference on human factors in computing systems.* pp 2215–2224.

[40] Sonderen EV, Sanderman R, Coyne JC (2013). Ineffectiveness of reverse wording of questionnaire items: Let’s learn from cows in the rain. PloS one.

[41] Li HW, Chung OKJ, Ho KYE, Chiu SY, Lopez V (2011). Coping strategies used by children hospitalized with cancer: An exploratory study. Psychooncology.

[42] Henseler J, Ringle CM, Sarstedt M (2015). A new criterion for assessing discriminant validity in variance-based structural equation modeling. J. Acad. Mark. Sci..

[43] Liu Q (2022). A Descriptive and phenomenological exploration of the spiritual needs of Chinese children hospitalized with cancer. Int. J. Environ. Res. Public Health.

[44] Carson V, Soeken KL, Grimm PM (1988). Hope and its relationship to spiritual well-being. J. Psychol. Theol..

[45] Zhang C, Lu Y (2020). The measure of Chinese religions: Denomination-based or deity-based?. Chin. J. Sociol..

[46] Rosenberg AR (2019). Hope and benefit finding: Results from the PRISM randomized controlled trial. Pediatr. Blood Cancer.

[47] Salimi H, Zadeh Fakhar HB, Hadizadeh M, Akbari M, Izadi N, MohamadiRad R, Akbari H, Hoseini R (2022). Hope therapy in cancer patients: A systematic review. Supportive Care Cancer.

